# Using the theory of planned behavior to explain birth in health facility intention among expecting couples in a rural setting Rukwa Tanzania: a cross-sectional survey

**DOI:** 10.1186/s12978-020-0851-1

**Published:** 2020-01-13

**Authors:** Fabiola V. Moshi, Stephen M. Kibusi, Flora Fabian

**Affiliations:** 1 Department of Nursing and Midwifery, the University of Dodoma, P.O. Box 259, Dodoma, Tanzania; 2 Department of Public Health, the University of Dodoma, P. O Box.259, Dodoma, Tanzania; 3 Department of Biomedical Sciences, the University of Dodoma, P. O Box.259, Dodoma, Tanzania

**Keywords:** Childbirth, Health facility, Intention, Attitudes, Perceived subjective norms, Behavior control

## Abstract

**Background:**

According to the theory of planned behavior, an intention to carry out a certain behavior facilitates action. In the context of birth in health facility, the intention to use health facilities for childbirth may better ensure better maternal and neonatal survival. Little is known on the influence of the domains of theory of planned behavior on birth in health facility intention. The study aimed to determine the influence of the domains of theory of planned behavior on birth in health facility intention among expecting couples in the rural Southern Highlands of Tanzania.

**Methods:**

A community based cross-sectional study targeting pregnant women and their partners was performed from June until October 2017. A three-stage probability sampling technique was employed to obtain a sample of 546 couples (making a total of 1092 study participants). A structured questionnaire based upon the *Theory of Planned Behavior* was used. The questionnaire explored three main domains of birth in health facility intentions. These three domains included; 1) attitudes towards maternal services utilization, 2) perceived subjective norms towards maternal services utilization and 3) perceived behavior control towards maternal services utilization.

**Results:**

The vast majority of study participants had birth in health facility intention. This included 499(91.2%) of pregnant women and 488(89.7%%) of their male partners partner. Only perceived subjective norms showed a significant higher mean score among pregnant women (M = 30.21, SD = 3.928) compared to their male partners (M = 29.72, SD = 4.349) t (1090) = − 1.965 at 95% CI = -0.985 to − 0.002; *p* < 0.049. After adjusting for the confounders, no intention to use health facility for childbirth decreased as the attitude [pregnant women (B = − 0.091; *p* = 0.453); male partners (B = − 0.084; *p* = 0.489)] and perceived behavior control [pregnant women (B = − 0.138; *p* = 0.244); male partners (B = − 0.155; *p* = 0.205)] scores increase among both pregnant women and their male partners.

**Conclusion:**

Despite the fact that majority of study respondents had birth in health facility intention, the likelihood of this intention resulting into practice is weak because none of the domains of theory of planned behavior showed a significant influence. Innovative interventional strategies geared towards improving domains of intention is highly recommended in order to elicit strong intention to use health facilities for childbirth.

## Plain English

According to the theory of planned behavior, an individual’s intention to engage in a certain behavior facilitates the practice of the behavior. Individuals are much more likely to intend to have healthy behaviors (use of health facility for childbirth) if they have positive attitudes about the behaviors, believe that perceived subjective norms (social pressure) are favorable towards those behaviors and believe they are able to perform those behaviors correctly. Also, a person’s intentions will be stronger when they have all three of the above than when they have only one. Research demonstrates that intentions matter – as the stronger a person’s intentions to use health facility for childbirth, the more likely that person will actually perform that behavior. However, it is important to remember that many outside factors and restrictions can prevent an individual from performing a behavior, even when they have an intention to do so.

This study used the theory of planned behavior to explain birth in health facility intention among expecting couples. The study tested the association between the predictors of intention (attitude, perceived subjective norms and perceived behavior control) as postulated in the theory of planned behavior and the intention. Three predictors of intention.

Majority of study respondents had intention to use health facility for childbirth. The intention to use health facility for childbirth among pregnant women was higher compared to the intention among their male partners. The reason for the difference could be routed from the traditional gender roles and responsibilities. Male partners are responsible in provision of financial support. They may find it expensive for their female partners to use health facility for childbirth than homebirths where they will not be required to pay for transport and staying allowance. When other factors were controlled only the perceived social pressure (perceived subjective norms) significantly influenced intention to use health facility for childbirth among pregnant women. When other factors were controlled among male partners only perceived behavior control showed a significant influence to birth in health facility intention. According to theory of planned behavior, birth in health facility intention was weak among both pregnant women and their male partners because only one predictor of intentions showed to be significant.

## Introduction

Most life-threatening complications which occur during childbirth are unpredictable which necessitate the use of skilled birth attendants [1]. The use of skilled birth attendants in developing countries have increased from 55% in 1990 to 65% in 2010. However, in South Asia and Sub-Saharan Africa, the use of skilled birth assistance remains as low as 45% in Sub-Saharan Africa and 49% in south Asia [2]. The use of skilled birth attendants in developing countries have increased from 55% in 1990 to 65% in 2010. The average use of skilled birth attendance in Tanzania in the period of 2010–2015 was 64% which is the same in Rukwa Region [3].

It is estimated that 293,300 maternal deaths occurred in 2013 worldwide [4]. The major causes of these deaths were; maternal hemorrhage (44,200 deaths), complications of abortion(43,700 deaths), maternal hypertensive disorders (29,300 deaths), maternal sepsis and other maternal infections (23,800 deaths) and obstructed labor (18,800 deaths) [4].

The majority of maternal deaths occurred in developing countries where there were 230,000 maternal deaths in 2013.Most of these deaths occurred in Sub Saharan Africa (62%) and South Asia (24%) which together account for 86% of maternal mortality worldwide [5]. The Tanzanian estimated maternal mortality ratio is 556/100,000 [3] meaning that for every 1000 live births in Tanzania, about 5 women die due to pregnancy related causes which amounts to 8000 maternal deaths per year. Maternal mortality ratio varies within Tanzania with the highest maternal mortality of 860 deaths per 100,000 live births [6] in Rukwa Region where the use of health facility for childbirth was only 31.1% of deliveries were assisted by skilled birth assistance [7].

The risk of a woman dying due to maternal causes in developing countries is high: one woman in every 76 deliveries [8]. Comparing the risk in Tanzania where one woman dies in every 44 deliveries, to the risk in Poland where one woman in every 22,100 deliveries dies from maternal causes [9], Tanzania ranks among the countries with the highest maternal mortality rates worldwide [10].

However, maternal health is more than the survival of pregnant women and mothers. Studies have found that for each woman who lost her life in the course of bringing another life, there are 20 others who suffer pregnancy-related illness or experience other severe consequences [11]. Such pregnancy–related illnesses include severe acute maternal illnesses and chronic illnesses. The severe acute illnesses are life threatening complications such as organ failure and lifesaving surgery which necessitate timely emergency obstetric care in a hospital so that they can survive [12].

The other type of maternal illness is chronic illnesses. These are conditions caused by the birthing process and while they are not life-threatening, they greatly impair the quality of life, such as fistula, uterine prolapse, and dyspareunia. Other disabilities are also called postpartum maternal morbidities and include urinary incontinence, hernias, hemorrhoids, breast problems, and postpartum depression [12].

Similar to maternal survival, the survival of neonates depends very much on investment in maternal care, especially access to skilled antenatal care, delivery and early postnatal services [13]. This is because 36% of all newborn deaths are due to severe infections which necessitate identification and treatment of infections during pregnancy as well as clean delivery practices [13]. Also, asphyxia (difficulty in breathing after birth) causes 23% of newborn deaths and can largely be prevented by improved care during labor and delivery [13].

The intensity of maternal and neonatal mortalities in these low resource settings are mostly contributed by the use of health facilities where there are skilled birth attendants. The practice e (use of health facility for child birth) is highly contributed to the intention to use health facility during pregnancy. According to the *Theory of Planned Behavior*, an individual will have the intention to perform a behavior when they evaluate it positively, believe that the important others think they should perform it, and perceive it to be within their own control [14]. The intention to use health facility for child is influenced by the way an individual evaluates birth in health facility. If they evaluate it positively, believing that important others think it is something worth doing and perceive they can do it then they will have the intention to use health facility for childbirth.

**Fig. 1:**
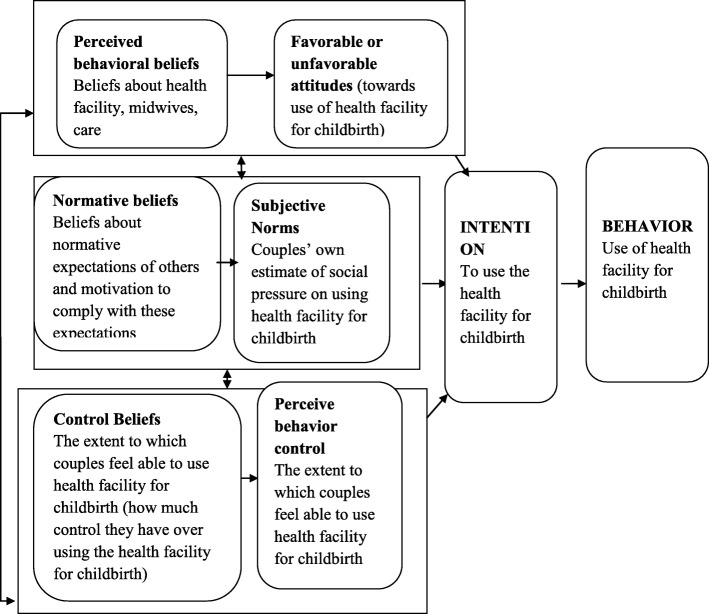
Theoretical model birth in health facility intention

An attitude toward a behavior refers to the degree to which a person has positive or negative feelings of the behavior of interest. It entails a consideration of the outcomes of performing the behavior [14]. A subjective norm refers to the belief about whether important others think he or she will perform the behavior. It relates to a person’s perception of the social environment surrounding the behavior [14]. Perceived behavior control refers to the individual’s perception of the extent to which performance of the behavior is easy or difficult [14] (see Fig. 1).

Previous studies have pointed out that among the causes of low use of health facility for childbirth are social demographic characteristics of expecting mothers (level of education, place of resident, parity etc) [15], low level of birth preparedness and low male involvement in birth preparedness [16–18]. Countries with good indicators in maternal and infant mortality have pregnancy related complications identified and managed early.

Little was known on the influence of the three domains of theory of planned behavior on birth in health facility intention. Therefore, this study reports the findings on the influence of the three domains of theory of planned behavior on intention to use health facility for childbirth.

## Methods

### Study design and setting

A community based cross-sectional study was conducted in Rukwa Region from June 1st - October 30th, 2017, among expecting couples from 45 villages in Rukwa Region in the Southern Highlands of Tanzania. The region had a population of 1,004,539 people; 487,311 males and 517,228 females. The forecast for 2014 was 1,076,087 persons with a growth rate of 3.5%. The region has the lowest mean age at marriage where males marry at the age of 23.3 years and 19.9 years for females and a fertility rate of 7.3 [19].

### Sampling method and sample size

#### Sampling technique

Two districts with the lowest rate of facility delivery in the Rukwa Region (Sumbawanga Rural District and Kalambo District) were purposively selected from the four districts of the region. Three staged multi-stage cluster sampling technique was used to obtain study participants. During first stage random samplings, all wards (12 wards of Sumbawanga Rural District and 17 wards of Kalambo District) in each district were listed and by the use of the lottery method of random sampling, five wards from Sumbawanga District and 10 from Kalambo District were picked. During second stage random sampling, all villages in the selected wards were listed and another simple random sampling was conducted to select 15 villages from Sumbawanga rural district and 30 villages from Kalambo District. The third stage sampling was a systematic sampling used to obtain households with pregnant women of 24 weeks gestation or less and living with a male partner. At each visited household, a female partner was interviewed for the signs and symptoms of pregnancy. A female partner who had missed her period for 2 months was requested to complete a pregnancy test. Those with positive tests who gave consent to participate were enrolled in the study. If a selected household had no eligible participants, the household was skipped and researchers entered into the next household.

#### Sample size calculation

The sample size for couples who were involved in the study was calculated using the following formula [20].

$$ \mathrm{N}=\frac{{\left\{Z\upalpha \surd \left[\uppi \mathrm{o}\left(1-\uppi \mathrm{o}\right)\right]+2\upbeta \surd \left[\uppi 1\left(1-\uppi 1\right)\right]\right\}}^2}{{\left(\uppi 1-\uppi \mathrm{o}\right)}^2} $$

Where:

n = maximum sample size.

Ζα = Standard normal deviation (1.96) at 95% confidence level for this study.

2β = standard normal deviate (0.84) with a power of demonstrating a statistically significant difference before and after the intervention between the two groups at 90%.

πο = Proportion at pre- intervention (Use of skilled delivery in Rukwa region 30.1%) [7].

π1_=_ proportion after intervention (Proportion of families which would access skilled birth attendant 51%) [7].

$$ \mathrm{n}=\frac{\left\{1.96\surd \right[0.301\left(1-0.301\right]+0.84\surd \left[0.51\left(1-0.51\right)\right]\Big\}{}^2}{{\left(0.6-0.51\right)}^2} $$

*n* = 162 couples + 10% =180.

A total of 546 couples were included in this study.

#### Data collection procedure

Data were collected using interviewers-administered questionnaires. Four trained research assistants (two from each district) were recruited, trained and participated in data collection. A questionnaire about domains of theory of planned behavior on birth in health facility intention was developed using the *Theory of Planned Behavior*. A pilot survey was done among ten (10) expecting couples from unselected village to test the reliability of the research tool. The questionnaire had two parts; i) social demographic characteristics ii) a Likert scale where respondents were supposed to strongly agree, agree, neutral, strongly disagree and disagree. The Likert response were scored as strongly agree = 5, agree = 4, neutral = 3, disagree = 2 and strongly disagree = 1.

There were three subparts of the statements in the Likert scale which focused on understanding of; i) attitudes towards maternal services utilization, ii) perceived subjective norms towards maternal services utilization iii) perceived behavior control towards maternal services utilization. Likert scale statements in the questionnaire were drafted differently between male and female respondents.

Attitude towards maternal services utilization among pregnant women, statements which were used were; “If I attend antenatal clinic four or more times, I am doing a good thing” “If I get vaccinated against tetanus toxoid, I am doing a good thing” “If I test for HIV during antenatal visits, I am doing a good thing” “If I test for Syphilis during pregnancy, I am doing a good thing” “If I attend for skilled birth attendant, I am doing a good thing” “If I attend for skilled postnatal services, I am doing a good thing. Attitudes towards maternal services utilization among male respondents were assessed using the following Likert statements; “If my wife attends antenatal clinic four or more times, she is doing a good thing” “If she gets vaccinated against tetanus toxoid, she is doing a good thing” “If she tests for HIV during antenatal visits, she is doing a good thing” “If she tests for Syphilis during pregnancy, she is doing a good thing” “If she is attended by a skilled birth attendant, she is doing a good thing” “If she attends for skilled postnatal service seven days after delivery, she is doing a good thing” and “If she utilizes the available maternal services, she will ensure good birth outcome”.

Perceived subjective norms towards maternal services utilization among pregnant women were assessed using the following Likert scale statements; “Important people to me think I should attend four or more antenatal visits” “Important people to me think I should get vaccinated against tetanus” “Important people to me think I should test for HIV during pregnancy” “Important people to me think I should get testes for syphilis during pregnancy” “Important people to me think I should use skilled birth attendants during childbirth” “Important people to me think I should attend postnatal care seven days after delivery” and “When it comes to maternal services utilization, I will do what the health care provider advise me to do”. Perceived subjective norms among male partners were assessed using the following Likert scale statements; “Important people to me, think my wife should attend four or more antenatal visits” “Important people to me, think she should get vaccinated against tetanus” “Important people to me, think she should test for HIV during pregnancy” “Important people to me, think she should get testes for syphilis during pregnancy” “Important people to me, think she should use skilled birth attendants during childbirth” “Important people to me, think she should attend postnatal care seven days after delivery” and “When it comes to maternal services utilization, she will do what the health care provider advise me to do”.

Perceived behavior control towards maternal services utilization among pregnant women were assessed using the following Likert statements; “For me to attend four or more antenatal clinics is simple and I can do it” “For me to get vaccinated against tetanus is simple and I can do it” “For me to be tested for HIV is trouble free and I can do it” “For me to be screened for STI such as syphilis is trouble free and I can do it” “For me to use skilled services for delivery is simple and I can do it” “For me to attend for postnatal checkups after seven days delivery is trouble free and I can do it” and “For me to use available maternal health services is simple and I can do so” Statements which were used among male partners were; “For my wife to attend four or more antenatal clinics is simple and she can do it” “For my wife to get vaccinated against tetanus is simple and she can do it” “For my wife to be tested for HIV is trouble free and she can do it” “For my wife to be screened for STI such as syphilis is trouble free and she can do it” “For my wife to use skilled services for delivery is simple and she can do it” “For my wife to attend for postnatal checkups after seven days of delivery is trouble free and she can do it” and “For my wife to use the available maternal health services, is simple and she can do so”.

#### Data processing and analysis

The data were checked for completeness and consistencies, were entered in to computer using statistical package IBM SPSS version 23. The analysis was done for each group (pregnant women and their male partners) separately. Scores on the domains of theory of planned behavior were treated as continuous scores. Descriptive characteristic on the continuous variables were used to generate mean scores, standard deviation. Independent t-test was used to compare mean scores between pregnant women and their male partners. For categorical variables, descriptive analysis was used to generate frequency distribution and cross tabulation was used describe the characteristics of the study participants. A chi-square test was used to test the relationship between socio-demographic characteristics and intention. All variables with *p*-value of 0.2 and below were used in bivariate logistic regression and multivariate logistic regression and ap-value below 0.05 was termed as a significant association.

## Results

### Results

#### Socio-demographic characteristics

A total of 546 couples were included in the study, with a response rate of 100%. The sample included 546 pregnant women (with gestational age of 24 weeks and below) and their partners. The mean age among the pregnant women was 25.57 years (SD = 6.810) and the mean age of their partners was 30.65 years (SD = 7.726). The majority of the couples were married (390, 71.4%), monogamous (469, 85.9%), live on less than 1 dollar per day (382, 70.0%), and receive their basic obstetric care services from dispensaries (452, 82.8). Ninety five percent of the cohort had completed primary school or less (Table 1).

**Table 1:** Socio-demographic characteristics of respondents

Character	Male (n_1_, %)	Female (n_2_, %)	Total (n_1_ + n_2_)
	(n_1_ = 546	(n_2_ = 546)	1092
Age (years)			
Less than 20	27 (4.9)	167 (30.6)	194 (17.8)
21 to 25	143 (26.2)	156 (28.6)	299 (27.4)
26 to 30	146 (26.7)	105 (19.2)	251 (23.0)
31 to 35	87 (15.9)	55 (10.1)	142 (13.0)
36 and above	143 (26.2)	63 (11.5)	206 (18.9)
Age at marriage (years)			
Less than 18	71 (13.0)	395 (72.3)	466 (42.7)
19 to 24	353 (64.7)	147 (26.9)	500 (45.8)
25 and above	122 (22.3)	4 (0.7)	126 (11.5)
Ethnic group			
Fipa	367 (67.2)	322 (59.0)	689(63.1)
Mambwe	118 (21.6)	120 (22.0)	238(21.8)
Others	61 (11.2)	104 (19.0)	165(15.1)
Education level			
Non-formal	155 (28.4)	230 (42.1)	385(35.3)
Primary school	353 (64.7)	299 (54.8)	652(59.7)
Secondary school or higher	38 (7.0)	17 (3.1)	55(5.0)
Income per day			
Less than 1 dollar	382 (70.0)	399 (73.1)	781(71.5)
More than 1 dollar	164 (30.0)	147 (26.9)	311(28.5)
Own radio			
Yes	308 (56.4)	253 (46.3)	561(51.4)
No	238 (43.6)	293 (53.7)	531(48.6)
Own mobile phone			
Yes	234 (42.9)	69 (12.6)	303(27.7)
No	312 (57.1)	477 (87.4)	789(72.3)
Adult female in the family			
None	315 (57.7)	318 (58.2)	633(58.0)
1 or more	231 (42.3)	228 (41.8)	459(42.0)
Covered by health insurance			
Yes	170 (31.1)	177 (68.9)	347(31.8)
No	376 (68.9)	369 (67.6)	745(68.2)
Health facility			
Dispensary	452 (82.8)	452 (82.8)	904(82.8)
Health center	94 (17.2)	94 (17.2)	188(17.2)
Approximately distance to reach to the health facility (Km)			
Less than 1	259 (47.4)	258 (47.3)	517(47.3)
1 to 5	232 (42.5)	233 (42.7)	465(42.6)
more than 5	55 (10.1)	55 (10.1)	110(10.1)

#### Mean scores of domains of theory of planned behavior compared between pregnant women and their male partners

When attitude mean scores were compared between pregnant women and their male partners, male partners had higher attitudinal mean score (M = 26.09, SD = 3.135), compared to pregnant women (M = 26.16, SD = 3.142), t(1090) = 0.366 at 95% CI = -0.303–0.442; *p* > 0.05 but the difference was not statistically significant. On perceived subjective norms mean scores, pregnant women had significantly higher mean score (M = 30.21, SD = 3.928) compared to their male partners (M = 29.72, SD = 4.349) t (1090) = − 1.965 at 95% CI = -0.985 to − 0.002; *p* < 0.049. On perceived behavior control, male partners had higher mean score (M = 30.47, SD = 3.668) compared to pregnant women (M = 30.45, SD = 3.771) t(1090) = 0.073 at 95% CI = 0.225 to − 0.425; *p* = 0.942.

#### Birth in health facility intention among pregnant women and their male partners

Majority of study participants 499(91.2%) of pregnant women and 488(89.7%) of their partners had intention to use health facility for childbirth.

#### The relationship between socio demographic characteristic and intention to use health facility for childbirth among pregnant women and their male partners

Among pregnant women characteristic of nearby health facility (*p* = 0.024) and owning a mobile phone (*p* = 0.018) were variables which influenced significantly birth in health facility intention (Tables 2 and 3).

**Table 2:** Distribution of participants by birth in health facility intention and factors affecting their intention (Chi-Square) among pregnant women

Variables	Have intention	No intention	X2	*p*-value
	n (%)	n (%)		
Age groups				
Less than 20	93 (92.1)	8 (8.9)		
21 to 25	138 (89.6)	16 (10.4)		
26 to 30	106 (93.8)	7 (6.2)		
31 to 35	68 (90.7)	7 (9.3)		
36+	93 (90.3)	10 (9.7)	1.672b	0.796
Ethnic group				
Fipa	299 (88.2)	40 (11.8)		
Mambwe	111 (99.1)	1 (0.9)		
Others	88 (92.6)	7 (7.4)	2.432a	0.296
Education level				
No formal	178 (91.8)	16 (8.2)		
Primary school	296 (91.4)	28 (8.6)		
Secondary school or higher	24 (85.7)	4 (14.3)	1.135b	0.567
Economic status				
Less than one dollar/dollar	351 (91.2)	34 (8.8)		
At least one dollar/day	147 (91.3)	14 (8.7)	.003c	0.959
Own a mobile phone				
Yes	146 (86.9)	22 (13.1)		
No	352 (93.1)	26 (6.9)	5.606c	0.018
Walking distance				
Less than 1 kilometer	238 (91.5)	22 (8.5)		
1–5 kilometers	206 (89.6)	24 (10.4)		
More than 5 kilometers	54 (96.4)	2 (3.6)	2.713b	0.258
Characteristic of health Facility				
Dispensary	417 (92.5)	34 (7.5)		
Health center	81 (85.3)	14 (14.7)	5.070c	0.024
Covered with health insurance				
Yes	157 (93.5)	11 (6.5)		
No	341 (90.2)	37 (9.8)	1.523c	0.217
Parity				
Para 0	104 (88.1)	14 (11.9)		
Para 1–4	297 (91.7)	27 (8.3)		
Para 5+	97 (93.3)	7 (6.7)	2.025b	0.363

When these factors were assessed among male partners, ethnic groups (*p* = 0.002), education level (*p* < 0.011) and covered with health insurance (*p* = 0.009) were variables which significantly influenced birth in health facility intention (Table 3)

**Table 3:** Distribution of participants by birth in health facility intention and factors affecting their intention (Chi-Square) among male partners

Variables	Have intention	No intention	X2	*p*-value
	n (%)	n (%)		
Age groups				
Less than 20	83 (89.2)	10 (10.8)		
21 to 25	124 (84.9)	22 (15.1)		
26 to 30	126 (91.3)	12 (8.7)		
31 to 35	61(92.4)	5 (7.6)		
36+	96 (93.2)	7 (6.8)	5.918a	0.205
Ethnic group				
Fipa	316 (90.5)	33 (9.5)		
Mambwe	114 (90.5)	12 (9.5)		
Others	60 (84.5)	11 (15.5)	12.779b	0.002
Education level				
No formal	178 (92.7)	14 (7.3)		
Primary school	292 (89.3)	35 (10.7)		
Secondary school or higher	20 (74.1)	7 (25.9)	9.107a	0.011
Economic status				
Less than one dollar/dollar	353 (89.4)	42 (10.6)		
At least one dollar/day	137 (90.7)	14 (9.3)	.220a	0.639
Own a mobile phone				
Yes	121 (90.3)	13 (9.7)		
No	369 (89.6)	43 (10.4)	.059a	0.807
Walking distance				
Less than 1 kilometer	229 (88.8)	29 (11.2)		
1–5 kilometers	214 (91.5)	20 (8.5)		
More than 5 kilometers	47 (87)	7 (13)	1.444a	0.486
Characteristic of health facility				
Dispensary	403(89.2)	49 (10.8)		
Health center	87(92.6)	7 (7.4)	.974a	0.324
Covered with health insurance				
Yes	152(84.9)	27 (15.1)		
No	338(92.1)	29 (7.9)	6.742a	0.009

#### The association between domains of theory of planned behavior and no intention to use health facility for childbirth

In crude odds ratio among pregnant women two domains (attitudes and perceived behavior control) showed a significant association with no intention to use health facility for childbirth. After controlling for the confounders (Own a mobile phone, characteristic of nearby health facility and covered with health insurance), among the domains of theory of planned behavior, no intention to use health facility for childbirth decreased as the attitude(B = − 0.091; *p* = 0.453) and perceived behavior control (B = − 0.138; *p* = 0.244) scores increase among pregnant women nevertheless the relationship was not statistically significant (Table 4).

**Table 4:** The association between domains of theory of planned behavior and no intention to use health facility for childbirth among pregnant women

Variable		OR	95%CI		p-value		AOR	95%CI		p-value
	B		Lower	Upper		B		Lower	Upper	
Attitude.	−0.105	0.9	0.816	0.993	0.04	−0.091	0.913	0.719	1.158	0.453
Perceived subjective norms.	−0.061	0.941	0.874	1.014	0.11	0.125	1.133	0.905	1.418	0.276
Perceived behavior control.	−0.088	0.916	0.846	0.991	0.03	−0.138	0.871	0.69	1.099	0.244
Own a mobile phone										
Yes		1					1			
No.	−0.032	0.969	0.504	1.862	0.92	0.86	1.062	0.546	2.063	0.86
Characteristic of health facility										
Dispensary		1					1			
Health center.	−0.043	0.958	0.433	2.121	0.92	0.036	1.037	0.462	2.327	0.93
Covered with health insurance										
Yes		1					1			
No	0.04	1.04	0.547	1.977	0.9	−0.007	0.993	0.517	1.908	0.983

Among male respondents, no significant association between the domains and no intention to use health facility for childbirth. After controlling of confounders among male partners (age group, ethnic group, education level and covered with health insurance), among the domains of theory of planned behavior, only attitude (B = -0.084; *p* = 0.489) and perceived behavior control (B = − 0.155; *p* = 0.205) scores showed a decrease in no intention to use health facility for childbirth as the scores increases (Table 5).

**Table 5:** The association between domains of theory of planned behavior and no intention to use health facility for childbirth among male partners

Variable		OR	95%CI		p-value		AOR	95%CI		p-value
	B		Lower	Upper		B		Lower	Upper	
Attitude	0.03	1.03	0.943	1.124	0.51	−0.084	0.92	0.726	1.165	0.489
Perceived subjective norms	0.001	1.001	0.94	1.066	0.98	0.132	1.141	0.901	1.445	0.274
Perceived behavior control	0.011	1.011	0.938	1.089	0.78	−0.155	0.857	0.675	1.088	0.205
Age groups										
Less than 20		1					1			
21 to 25	−0.68	0.507	0.234	1.094	0.08	−0.704	0.495	0.224	1.095	0.083
26 to 30	−0.875	0.417	0.184	0.945	0.04	−0.803	0.448	0.194	1.038	0.061
31 to 35	−1.895	0.15	0.033	0.679	0.01	−1.881	0.152	0.033	0.7	0.016
36+	−0.267	0.766	0.351	1.67	0.5	−0.263	0.769	0.339	1.742	0.528
Ethnic group										
Fipa		1					1			
Mambwe	0.663	1.941	1.046	3.602	0.04	0.723	2.06	1.069	3.972	0.031
Others	0.487	1.628	0.734	3.61	0.23	0.714	2.041	0.878	4.744	0.097
Education level										
No formal		1					1			
Primary school	0.141	1.151	0.642	2.065	0.64	0.243	1.275	0.691	2.351	0.437
Secondary school or higher	−1.055	0.348	0.045	2.712	0.31	−0.613	0.542	0.066	4.428	0.567
Covered with health Insurance										
No		1					1			
Yes	−1.026	0.358	0.172	0.748	0.01	0.006	0.343	0.16	0.734	0.006

## Discussion

An individual’s intention to perform a certain behavior is influenced by an individual’s attitude towards the behavior, the perceived subjective norms and the perceived behavior control. In this study the behavior of interest was birth in health facility. According to the theory of planned behavior, birth in health facility intention is influenced by the attitude the individual has about birth in health facility, the perceived subjective norms this particular individual has and the perceived control on performing the behavior [13].

This study found that majority of study respondents (91.2% of pregnant women and 89.7% of their male partners) had birth in health facility intention. A similar findings has been reported by a study done by Creanga et al. [21] . The intention among male partners was lower than the intention of their female partners. The reason could be that male partners avoid financial implications associated with health facility childbirth. Avoidance of financial responsibility may be attributed to gender norms which influence men not to prioritize access to skilled birth attendance as pregnancy and childbirth are perceived to be women’s affairs [22].. The access to maternal and child health in Tanzania is free [23] but there are some hidden cost associated with decision to choose health facility for childbirth. The costs include, transport costs, costs to procure birthing items and the cost of staying in health facility. Male partners may opt for home childbirth assisted by unskilled attendance to avoid financial implications and hence lower proportion of intention to use health facility for childbirth.

Low risk perception towards pregnancy and childbirth could be another reason for some male partners to prefer home childbirth over health facility childbirth [22]. When they perceive pregnancy and childbirth is a normal process and not associated with risks may lower their intention to use health facility for childbirth.

Another reason could be low knowledge about risks associated with pregnancy and childbirth among expecting couples in this community [24]. Men have low knowledge on risks which are associated with pregnancy and childbirth compared to female [24]. Their low knowledge on risks associated with pregnancy and childbirth may lower their intention to use health facility for childbirth. Choosing health facility for childbirth ensure timely intervention in case of any complication which may occur during childbirth.

Despite the fact that, the majority of study participants had intention to use health facility for childbirth, their intention was weak. According to theory of planned behavior, the intention to engage into a behavior is predicted by three variables-the attitudes, perceived subjective norms (socio pressure) and perceived behavior control. A person’s intentions will be stronger when all domains of intention have significant influence to the intention [25]. Research demonstrates that intentions matter – as the stronger a person’s intentions to have a healthy behavior, the more likely that person will actually perform that behavior [25].

The study found that after adjusting for confounders, the attitudinal scores and perceived behavior control scores among both pregnant women and their male partners decreases when there was no intention to use health facility for childbirth. Despite the fact that the relationship was not statistically significant, the study showed a decrease odd when the attitudinal scores and perceived behavior scores increases. This means that when both pregnant women and male partners have increased attitude towards maternal services utilization their likelihood to intend to use health facility for childbirth increases. According to theory of planned behavior, the attitude is influences by the evaluation about the benefit of the behavior [14]. Also, there was a decreased odd of no intention to use health facility for childbirth as the perceived behavior control scores increase among both pregnant women and their male partners. This means that when couples believe they are capable of engaging into a behavior, the likelihood of not intending to participate into the behavior decreases.

On contrary, Odds of no intention increases with increase in perceived subjective norms scores. This means that the perception of social pressure to support use of maternal services increase the intention not to use health facility for childbirth. In other words, social pressure does not increase the intention to use health facility for childbirth. This is contrary to what was postulated by the theory of planned behavior where perceived subjective norms is among the domains which increase intention to engage into a behavior [14].

This study found that none of the domains of theory of planned behavior showed a significantly influenced on birth in health facility intention among both pregnant women and their male partners. This means that the intention to engage into the behavior is weak and may not be translated to action This is translated in the real practice of low use of health facility for childbirth in this community where more than 69% of birth occur outside health facility assisted by unskilled personnel [7]. The finding is in contrast with a similar study done in Ethiopia where all predictors of intention significantly influenced the intention [26]. The difference in finding is due to differences in place of receiving skilled maternal care. While this study was about birth in health facility intention, the study in Ethiopia was about intention in maternity home.

In addition to the existing interventions in Tanzania such as increasing number of facilities and removed financial barriers in accessing maternal services, behavior theory integrated interventions to address deep seated predictors of health seeking behaviors is highly recommended.

## Conclusion

Despite the fact that majority of study respondents had birth in health facility intention, the likelihood of this intention resulting into practice is weak because none of the domains statistically influenced the intention to use health facility for childbirth. Innovative interventional strategies geared towards improving domains of theory of planned behavior among expecting couples are highly recommended in order to improve birth in health facility intention.

## Data Availability

Data set is available and can be shared on request.

## Abbreviations

AOR: Adjusted Odds Ratio
HIV: Human immunodeficiency virus
IBM: International Business Machines
SPSS: Statistical Package for the Social Sciences
UNICEF: United Nations Children’s Fund
WHO: World Health Organization
